# Lost in Translation: The Role of Interpreters on Labor and Delivery

**DOI:** 10.1089/heq.2020.0016

**Published:** 2020-09-30

**Authors:** Margot Le Neveu, Zackary Berger, Marielle Gross

**Affiliations:** ^1^ Department of Gynecology and Obstetrics, Johns Hopkins Hospital, Baltimore, Maryland, USA.; ^2^ Johns Hopkins University Berman Institute of Ethics, Baltimore, Maryland, USA.; ^3^ Johns Hopkins Bloomberg School of Public Health, Baltimore, Maryland, USA.; ^4^ Department of General Internal Medicine, Johns Hopkins School of Medicine, Baltimore, Maryland, USA.; ^5^ Department of Obstetrics and Gynecology, Greater Baltimore Medical Center, Baltimore, Maryland, USA.

**Keywords:** language barrier, interpreters, obstetric, disparities

## Abstract

During the Coronavirus (COVID-19) pandemic, in-person interpreters have been deemed “nonessential,” and thus eliminated to minimize viral exposure and conserve personal protective equipment. Considering alarming patterns of interpreter underuse, we evaluate how substitution for remote modalities (telephone or video) may exacerbate existing inequalities for patients with limited English proficiency. The inherent intimacy, dynamic physicality, and cultural nuances of labor and delivery pose unique communication challenges. Using clinical scenarios, we illustrate the vital role interpreters have in providing accessible obstetric care. We argue that eliminating in-person interpreters in this setting is not justified by COVID-related harms given the potential to exacerbate underlying health disparities.

## Introduction

The Coronavirus (COVID-19) pandemic has required elimination of “nonessential” members of the clinical team, including interpreters, to decrease exposure and personal protective equipment utilization. This well-intended infection control measure may reinforce disparities. Limited English proficiency (LEP) can contribute to clinically meaningful and potentially morbid misunderstandings. This is substantiated in the obstetric setting, as illustrated by a confounder-adjusted analysis of parturients whose first language was not English (*n*=11,419) who experienced approximately twice as many severe pelvic lacerations (adjusted relative risk: 2.02; 95% confidence interval: 1.34–3.04) and significantly higher rates of primary cesarean delivery (*p*=0.011).^1^

During the current pandemic, there was a delay in testing family members for a Spanish-speaking patient in Michigan exhibiting COVID-19 symptoms, and patients in New York City have been missed, confused, left without advocates, and received substandard care just because of language barriers.^2,3^ We consider the impact eliminating in-person interpreters may have in providing high-quality equitable obstetric care contextualized by the following two scenarios.

### Scenario 1

The status board shows a chief complaint: “decreased fetal movement,” and you wheel the ultrasound and video translation tablet stand into the room. With repositioning to respect patient privacy, two towers now stand between you. The anxiety in the room mounts while you wait for the video interpreter connection. By the time you use bedside ultrasound to search for, and ultimately fail to find, a fetal heartbeat, the patient is in tears. You share your findings, but the statement is complicated, and the interpreter has difficulty hearing over the weeping. A dialogue that should be occurring with the patient is instead a series of clarifications as the interpreter interjects “one moment, interpreter requires repetition” multiple times until the statement is a mere skeleton of the original. Despite your best attempts trying to communicate bad news through a screen, you are alone in the room with a patient with whom you do not share a common language.

When will the in-person interpreter arrive…

### Scenario 2

A patient is screaming in labor while you fumble to place the interpreter phone in a mutually accessible area. Without passing the phone back and forth, neither you nor the patient can hear what is said. The baby's heart rate is decelerating, and the mother is pushing ineffectively. To everyone's relief, the interpreter arrives, a familiar face on the floor. She immediately grabs one of the patient's legs and coaches her. Deliberately counting, encouraging, and relaying your feedback in real time to the patient brings a perspective that the phone interpreter just could not provide. The patient delivers a beautiful baby, and our interpreter is the first team member to congratulate her.

Who would you have wanted *in the room*?

## In-Person Versus Remote Interpretation on Labor and Delivery

Implementing the use of professional interpreters has been shown to reduce disparities, improve clinical outcomes, enhance pain control, and improve patient satisfaction.^4,5^ In-person interpreters convey both verbal and critical nonverbal patient expressions and are often engaged members of the care team. We know that continuous support during labor improves obstetric outcomes,^6^ and for patients with language barriers, an in-person interpreter may be similarly essential. They provide invaluable context and nuance during the admission and consent process, coach patients in effective pushing, guide mothers through cesarean deliveries, and offer explanation if a baby is unexpectedly taken to the neonatal intensive care unit.

In-person interpretation is an expensive resource that is unfortunately limited at baseline. The availability of remote interpretation (telephone or video), however, does not justify its use as a substitute in all settings. There are some circumstances in which remote methods are preferred,^7,8^ but many patients, interpreters, and providers express preference for in-person encounters.^9^ Although current evidence does not conclusively identify a superior mode of interpretation in all settings,^10^ remote tools can be difficult to use and depend on adequate wi-fi, variable wait time fluctuating with language availability, and inconsistent interpreter skillset. A comprehensive pilot study comparing remote and in-person interpretation modalities at the Cambridge Health Alliance identified situations poorly suited for telephone interpretation, including commotion in the room, procedures, and trying to communicate with multiple people,^7^ all common features of labor and delivery. We consider this issue from multiple perspectives.

### Patient perspectives

Language barriers impact health outcomes and patient satisfaction,^11–13^ and communication breakdown may leave permanent scars. Technical and logistical challenges of remote technology may prompt reduction of clinical communication to the bare minimum.^7^ This lack of nuance increases confusion among patients, leaves unanswered questions, undermines of trust, and may contribute to lasting traumatic memories of obstetric care.^13^ Cultural, societal, and family norms of childbirth are unique and important to elicit. Pregnant women who spoke English less than “very well” were more likely to report not having an active role in decision-making with respect to their labor.^12^ Postpartum women with LEP expressed a strong preference for in-person interpretation compared with remote modalities.^9^ In a randomized trial among patients seen in a tertiary care emergency department, a setting comparable with labor and delivery triage, those allocated to the in-person arm were significantly more satisfied with their interpreter service, significantly more satisfied with their physicians, and rated their ability to communicate significantly higher than the patients allocated to telephone or *ad hoc* interpretation (*p*<0.001).^14^ Although some patients identify the benefit of anonymity with telephone interpretation in settings where the need for privacy is high and the nature of communication is straightforward, communication quality and effectiveness was overall improved with in-person interpreters.^14,15^

### Interpreter perspectives

Interpreters are uniquely equipped to assess how well information has been communicated with all parties involved. In-person interpreters enable a genuine dialogue by conveying patients' desires, questions, and fears. To develop trust in complex dynamic settings, such as when more than two people are in a room, as is commonly the case during labor and delivery, most interpreters preferred in-person encounters.^16^ They can act as patient advocates, empower cross-cultural physician–patient relationships, and directly contribute to the therapeutic bond. Although interpreters were equally satisfied with remote and in-person modalities for straightforward information exchange, they found in-person exchange was more satisfactory for establishing rapport and facilitating physician understanding of patients' cultural background.^17^ Interpreters cite that being in-person to “read the room” and assess body language is key for effective and accurate communication.^16^

### Provider perspectives

Providers were more likely to indicate improved understanding of patients' cultural beliefs with in-person interpreters compared with video interpretation.^18^ Similar to interpreters, providers rated in-person services significantly higher than video and phone resources, citing frequent technical difficulties while using remote methods.^9^ Participating in nonverbal communication, serving as a culture broker/patient advocate, and being able to gather more accurate information from patients were cited as benefits of in-person services.^7^ As providers are principally responsible for orchestrating the involvement of interpretation services, their perspective is paramount.

## Background of Underutilizing and Devaluing Professional Interpreters

Eliminating in-person interpreters in favor of remote services during the COVID-19 pandemic devalues in-person interpreters and is consistent with underutilization trends. Despite availability of professional interpreters and what we know about the relationship between language barriers and clinical outcomes, certified interpreters are used for <20% of patients who would benefit.^19–22^ Title VI of the Civil Rights Act mandates health care organizations that receive federal funding must provide patients access to language services,^23^ but patients report often just “getting by.”^19,22^

Residents frequently rely on their own limited language skills or *ad hoc* interpreters such as family members or staff.^5^ Despite availability of interpreters, providers cite time constraints and organizational-level considerations, including interpreter and telephone availability, among the factors limiting interpreter use.^21^ Using *ad hoc* or untrained interpreters increases communication errors such as omission of information, word addition or substitution, and editorialization.^24^ Universal masking in clinical settings is an additional physical barrier to relationship building^25^ and clear communication that may be particularly significant across language barriers with elevated importance of facial expression. Eliminating in-person interpreters as nonessential in the care of patients with LEP during COVID-19 compromises communication and sacrifices a critical patient advocate, exacerbating disparities in an at-risk population.

## Conclusion and Call to Action

Wholesale elimination of in-person interpreters during COVID-19 is a mechanism by which health crises hit our vulnerable patients the hardest. The concurrent restriction of most in-person visitors and universal masking requirements exacerbate potential harms and heighten the need for critical patient advocates. Use of remote instead of in-person interpreters is a compromise in quality of care that we should not be willing to accept on our patients' behalf. Table 1 identifies labor and delivery encounters for which in-person interpretation should be considered essential during COVID-19 and beyond.

**Table 1.** Recommendations for Use of Interpretation Services on Labor and Delivery

**In-person interpretation** (if available)
Informed consent conversations
Discussions regarding death and dying:
• Breaking bad news, fetal loss, and terminal illness
Procedures:
• Starting labor induction
• Transfer to and from the OR for procedures with sedation
• Vaginal delivery
• Cesarean delivery
• Epidural placement
Psychiatric or psychosocial evaluations
• Suicide attempts, capacity assessment, and postpartum depression screen
• Intimate partner violence screening and assessments
• Discussions with social work and child welfare services
Initial lactation consultation
Counseling regarding adverse events and medical errors
Discharge instructions
**Remote interpretation** (phone or video)
Remaining clinical encounters including but not limited to:
• Initial triage evaluation
• Introduction of new staff with change of shift
• Pitocin adjustments
• Medication administration
• Pelvic examinations, including cervical examinations (phone preferred)
• Communicating with patient family members, unless regarding aforementioned subjects
• Repositioning in bed
• Routine outpatient obstetric and gynecological care

Provides examples of high-risk clinical encounters on labor and delivery for which use of an in-person interpreter can be considered during the COVID-19 pandemic and beyond.

COVID-19, Coronavirus.

Although we advocate for broader use of interpreters overall, we acknowledge that not all encounters equally benefit from in-person services. Optimal use of available interpretation modalities, including the decision to invoke an in-person interpreter in specific circumstances, requires judicious clinical judgement.

Minimizing health disparities among patients with LEP requires linguistic support. The pandemic has highlighted the need to re-examine and reprioritize in-person interpreter use in clinical settings. Going forward, we encourage institutions and governing bodies to promote this effort accordingly.

## Abbreviations Used

COVID-19: Coronavirus
LEP: limited English proficiency
